# Cognitive Flexibility and Advice Network Centrality: The Moderating Role of Self-Monitoring

**DOI:** 10.3389/fpsyg.2018.01947

**Published:** 2018-10-09

**Authors:** Saiquan Hu, Zhou Zhong, Jin Zhang, Xiaoying Zheng

**Affiliations:** ^1^Sloan School of Management, Massachusetts Institute of Technology, Cambridge, MA, United States; ^2^Institute of Education, Tsinghua University, Beijing, China; ^3^School of Management, Jinan University, Guangzhou, China; ^4^Department of Marketing, School of Business, Nankai University, Tianjin, China

**Keywords:** cognitive flexibility, self-monitoring, advice network centrality, advice network generation theory, power

## Abstract

This study examined the role of individuals’ cognitive flexibility and self-monitoring in shaping their workplace advice network centrality. Drawing on advice network generation theory, we hypothesized a positive relationship between cognitive flexibility and advice network centrality, and a moderation effect of self-monitoring on this relationship. Then, we collected two time-points data from insurance salesmen to test the hypotheses. As predicted, cognitive flexibility was positively associated with advice network centrality. Furthermore, this positive relationship was only significant for low self-monitoring individuals, but not for high self-monitoring individuals. These findings indicated that individuals with high cognitive flexibility were more likely to have central positions in the advice network; however, this effect was attenuated as their self-monitoring increased.

## Introduction

Individuals pursue power to influence others in the workplace (Sparrowe and Liden, 2005). One approach to acquire this power is to become an advice source for others and occupy centrality in the advice network (Sparrowe et al., 2001). Previous literature has shown that individuals with the personality traits, such as proactive, charismatic, and extroversive are more likely to occupy advice network centrality. The underlying reason is that they are more willing to help and share resources with advice seekers (Klein et al., 2004; Balkundi et al., 2011; Yang et al., 2011). However, on deciding to build a relationship with potential advice source, advice seekers not only value the provided assistance and shared resources, but also value for complex advice forms, such as generating novelty solutions, reformulating complex problems, and offering assertive validations (Cross et al., 2001). Therefore, in order to fulfill the potential complexities of demands from advice seekers, it is imperative for those who want to become advice sources and occupy central positions in the advice network to think and behave with cognitive flexibility (Martin and Anderson, 1998).

Cognitive flexibility, on the cognitive level, refers to an individual’s awareness of alternative solutions in any given situation and willingness to be flexible (Martin and Anderson, 1998). Moreover, on the behavioral level, it refers to the ability of shifting mental sets and thoughts in order to perceive and respond to changing demands in different ways (Eslinger and Grattan, 1993; Dajani and Uddin, 2015). Previous research on the consequences of cognitive flexibility shows that it increases individual’s creativity and resilience to negative events (Genet and Siemer, 2011; Chen et al., 2014). However, little is known about its effect on shaping an individual’s advice network position. Therefore, this study explored the relationship between cognitive flexibility and advice network centrality.

Advice network centrality is determined by the number of actual advice relationships that a group of people are willing to develop with a focal people (Freeman, 1979). Therefore, a focal individual can obtain advice network centrality by converting people in his or her network from potential advice seekers to become actual ones. Even if a focal person’s cognitive flexibility might motivate potential advice seekers to initiate relationships, the person’s potential value will be perceived by advice seeker as high volatility and low accessibility if the focal person’s advice giving behaviors are situation-dependent. Self-monitoring is an effective indicator of a person’s situation-dependent behavior. It refers to the extent to which individuals monitor, regulate, adjust, and control the public image of the self in interpersonal relationship development (Snyder, 1974; Shaffer et al., 2015). Prior research indicates that self-monitoring has negatively moderation effect on the relationship between personality and performance (Barrick et al., 2005). This study further investigated whether self-monitoring can negatively moderate the effect of cognitive flexibility on advice network centrality.

In the following section, the advice network generation theory is applied to explain why cognitively flexibility has a positive effect on advice network centrality and how self-monitoring mitigates this effect.

## Theoretical Framework and Hypotheses Development

### Advice Network Generation Theory

According to advice network generation theory, advice seekers are self-interest orientated. They weigh potential benefits and costs associated with seeking advice from a focal person (Nebus, 2006). In an advice relationship, the benefits refer to using the focal person’s expert knowledge, skills, and resources to solve problems; whereas the costs refer to the risks of appearing inferior or incompetent and feeling embarrassed or uncomfortable (Borgatti and Cross, 2003; Klein et al., 2004; Schulte et al., 2012). When advice seekers calculate cost and benefit in developing an advice relationship, a focal person’s likelihood for being sought for advice in a network depends on both the potential benefits one can provide and the potential costs one can incur (Erdogan et al., 2015). In other words, those who are expected to yield more benefits than costs during advice interactions are a potentially valuable advice source for advice seekers and are more likely to occupy advice network centrality (Nebus, 2006).

## Effect of Cognitive Flexibility on Advice Network Centrality

Cognitive flexibility increases one’s likelihood to become advice sources and advice network centrality in two ways. First, cognitive flexibility enables individuals to respond to a stimulus with accuracy and efficiency, and exhibit behaviors such as multitasking, novelty generation, and flexible problem-solving (Ionescu, 2012). Therefore, when processing complex problems raised by advice seekers, cognitively flexible individuals can absorb and process information flexibly by switching mental sets from one scenario to another, then produce diverse ideas and contemplate solution alternatives (Johnco et al., 2013). This process not only generates direct solutions but also reformulates the complex problems to enlighten advice seekers. Second, cognitive flexibility represents a vital component of communication competence (Curran and Andersen, 2017). Individuals who are cognitively flexible are more responsive, assertive, and empathetic in communication, and are more likely to achieve social interaction goals in various situations (Martin and Anderson, 1998). They are also adept at taking others’ perspectives and giving assertive feedback to advice seekers who require validation and confirmation about their tasks (Cross et al., 2001). By doing so, they mitigate advice seekers’ concerns for embarrassment or uncomfortableness.

In summary, cognitively flexible individuals have the ability to generate novel solutions that help others solve complex tasks and engage in comfortable communication that prevent or alleviate others’ embarrassment. These features render cognitively flexible individuals potentially valuable advice sources because they yield more benefits while incurring lower costs for advice seekers. We therefore proposed Hypothesis 1.

**H1:** *Cognitive flexibility is positively related to advice network centrality.*

In the following subsection, we further argued that how self-monitoring moderates the positive relationship between cognitive flexibility and advice network centrality.

## Moderating Effect of Self-Monitoring

Even though a cognitively flexible individual can be identified as a potentially valuable advice source, the advice seekers’ possibility of actually seeking advice from that person depends on the accessibility of that person’s potential value. When a cognitively flexible individual shows a strong willingness to use such cognitive ability to solve others’ problems across different situations (Nebus, 2006; Erdogan et al., 2015), the advice seeker will pick up that cue and mark that person as a capable and accessible source of advice. Therefore, self-monitoring, which features a behavioral inconsistency across situations (Snyder, 1974; Barrick et al., 2005), is regarded as an appropriate cue for advice seekers to evaluate the adviser’s accessibility value.

High self-monitoring individuals are status-enhancement motivated. They often act differently across situations and over time in order to gain status and impress others (Premeaux and Bedeian, 2003; Grieve, 2011; Oh et al., 2014). Thus, when cognitively flexible individuals have high self-monitoring, it is a situation-dependent question that to what extent they want to use their cognitive ability to help others. In other words, a person with high cognitive flexibility may exhibit a wide variation of accessibility to their advice seekers given their self-monitoring level. Specifically, when the provision of advice does not enhance status (e.g., becoming a role model) or incurs the risk of hurting their public image (e.g., reputation loss resulting from failure of their suggestions), these people may choose not to engage in an advice relationship. Consequently, advice seekers will find it difficult to accurately predict such people’s responses and evaluate their accessibility in providing help, which increases the cost of seeking advice from such people.

In contrast, low self-monitoring individuals are guided by a core set of altruistic values and beliefs. Their attitudes, intentions, and behaviors are more stable across various situations and over time (Premeaux and Bedeian, 2003; Oh et al., 2014). Thus, their advice giving behaviors are not situation-dependent. This behavior consistency feature of such people allows advice seekers to form more accurate inferences about their responses and values and evaluate them as more accessible. This, in turn, makes such people good targets for seeking advice (Funder, 1995).

In summary, advice seekers will prefer seeking advice from cognitively flexible individuals with low self-monitoring, but refrain from seeking advice from those with high self-monitoring due to the cost associated with the high uncertainty and low accessibility of their potential values. Therefore, we proposed Hypothesis 2.

**H2:** *Self-monitoring moderates the relationship between cognitive flexibility and advice network centrality. Specifically, cognitive flexibility is positively related to advice network centrality when individuals’ self-monitoring is low. The positive relationship is not significant when individuals’ self-monitoring is high.*

In order to test the proposed two hypotheses, we chose salesmen as participant and designed a two time points survey. The detailed information is presented in the following section.

## Materials and Methods

### Participants and Procedures

Participants for this study consisted of 132 salesmen at an insurance company in Northeast China. These participants were selected for two reasons. First, insurance salesmen are required to be skillful in generating new ideas and reframing problems in customer engagement. Second, salesmen have strong motivation to seek advice from each other to deal with complex customer demand as the company reward teamwork by making each salesman’s salary contingent on the performance of the whole team. This study has been approved by the Ethic Review Committee of Tsinghua University.

In order to reduce common-method bias (Podsakoff et al., 2003), we collected data at two time points with 1 month interval. At Time 1, all participants completed measures of cognitive flexibility and self-monitoring, and reported demographic information. Eight participants who reported the same score across all items were removed from further analysis. Of these remaining 124 participants, 77% were female. Their average age is 45.77 (*SD* = 7.39) and their average work experience is 7.97 (*SD* = 2.71). At Time 2, the 124 salesmen participants in the first round survey were asked to complete a questionnaire to assess advice network centrality. Specifically, each of salesman reported the advice relationships with other salesmen. Six participants who nominated all other salesmen as advice sources were removed from further analysis. The final response rate reached 89%, meeting the required response rate for network data investigation (Sparrowe et al., 2001).

All participants in the two surveys were invited to a meeting room to receive an envelope that included a questionnaire and a notebook as a reward. Participants were notified about consent-related details both orally and in writing, before signing the informed consent form and commencing the questionnaire. They were asked to seal the questionnaire in an envelope and return it on site once completed.

### Measures

All measurements used in this research were originally in English and subsequently translated into Chinese following the back-translation process (Brislin, 1970).

#### Cognitive Flexibility

Seven items adapted from Martin and Rubin’s (1995) 12-item cognitive flexibility scale were used (the other five items of this scale were not selected because they are not applicable to insurance sales setting). Participants rated their agreement with these items on a 6-point Likert scale from 1 (strongly disagree) to 6 (strongly agree). A sample item was “I am capable of overcoming the difficulties in life that I face.” The answers to all the reverse items were reversely coded to compute the score of cognitive flexibility, with higher score representing higher cognitive flexibility. The Cronbach’s α for this scale was 0.72.

#### Self-Monitoring

The 13-item revised self-monitoring scale from Lennox and Wolfe (1984) was used. Participants indicated to what extent they agree with these items on a 5-point Likert scale from 1 (strongly disagree) to 5 (strongly agree). A sample item was “Once I know what the situation calls for, it is easy for me to regulate my actions accordingly.” The Cronbach’s α for this scale was 0.84.

#### Advice Network Centrality

To reduce self-report bias (Calloway et al., 1993), a whole network roster method rather than self-reported measurement was used to assess the advice relationship. Specifically, participants first read the description selected from Sparrowe et al. (2001) “people from whom you seek advice or help concerning any task problems”; then they were presented with the names of all the salesmen and were asked to choose the names that fit the description. After obtaining all participants’ reported advice relationships, the relationships were then arranged in a matrix format, in which cell *X*_ij_ corresponded to i’s advice relation to j as reported by i. For example, if i reported going to j for advice, then cell *X*_ij_ in the advice matrix was coded as 1; otherwise the cell was coded as 0. This matrix form data was then entered into UCINET software to calculate network centrality, with 0 representing lowest centrality and 1 representing highest centrality. Following Sparrowe and Liden’s (2005) suggestion, the in-degree network centrality of each participant was measured because this index captures the extent to which a certain person in a network is nominated by others as an advice source.

#### Control Variables

Individual level demographics such as tenure, education (1 = below high school, 2 = high school, 3 = college, and 4 = above college), and gender (female = 0, male = 1) were included as controls since a previous study has demonstrated those variables are associated with the advice network centrality (Klein et al., 2004).

## Results

### Descriptive and Correlational Analyses

All data of variables were analyzed using SPSS version 21.0. **Table 1** shows the means, standard deviations, and correlations of the variables. The mean value of advice network centrality is 0.61. Gender was negatively correlated with advice network centrality and cognitive flexibility was positively correlated with advice network centrality (*r* = 0.199, *p* = 028); however, self-monitoring was not significantly related to advice network centrality (*r* = 0.131, *p* > 0.1).

**Table 1:** Means, standard deviations, and correlations among variables.

Variables	Mean	*SD*	1	2	3	4	5
(1) Tenure	7.93	2.74					
(2) Education	1.50	0.64	−0.187^∗^		.		
(3) Gender	0.23	0.42	−0.203^∗^	0.143			
(4) Cognitive flexibility	4.91	0.61	0.186^∗^	−0.021	−0.111		
(5) Self-monitoring	4.06	0.44	−0.016	−0.102	−0.041	0.041	
(6) Advice network centrality	0.61	0.25	−0.043	0.071	−0.182^∗^	0.199^∗^	0.131

*^∗^p < 0.05.*

### Regression Analysis and Hypotheses Testing

Advice network centrality that was measured in the second time point was treated as the dependent variable, the other variables that were measured in the first time point, including controls, independent variable (cognitive flexibility), moderator (self-monitoring), and the interaction term of independent variable and moderator were entered into the model in a step-wise fashion (see Model 1–4 in **Table 2**). The ordinary least square (OLS) regression models were used to test the two hypotheses of this study as the dependent variable was normally distributed and the independent variable and moderator was not correlated. The result showed that cognitive flexibility was significantly positively related to advice network centrality (β = 0.048, *t* = 2.15, and *p* = 0.03, see Model 2 in **Table 2**). Therefore, it supported Hypothesis 1 that cognitive flexibility had a positive effect on advice network centrality. In addition, gender was significantly negatively related to advice network centrality, indicating that women were more likely to have central positions in advice network than men.

**Table 2:** Ordinary least square models with advice network centrality as dependent variable.

	Advice network centrality			
Control variables	Model 1	Model 2	Model 3	Model4
Tenure	−0.006	−0.009	−0.008	−0.008
Education	0.033	0.032	0.037	0.042
Gender	−0.121^∗^	−0.113^∗^	−0.112^∗^	−0.091
**Independent variable**				
Cognitive flexibility		0.048^∗^	0.047^∗^	0.053^∗^
**Moderator**				
Self-monitoring			0.028	0.038
**Interaction term**				
Self-monitoring × Cognitive flexibility				−0.059^∗∗^
*R*^2^	0.044	0.081	0.093	0.164
Δ*R*^2^	0.019	0.049	0.054	0.119

*^∗^p < 0.05,^∗∗^p < 0.01.*

Since adding interaction term usually causes multi-collinearity problems in the OLS model, we followed Hair et al. (1998) method to calculate mean-centered cognitive flexibility and self-monitoring before testing the moderating effect of self-monitoring. According to Model 4 in **Table 2**, self-monitoring significantly negatively moderated the effect of cognitive flexibility on advice network centrality (β = -0.059, *t* = -3.08, and *p* < 0.01). Thus, it supported Hypothesis 2 that self-monitoring negatively moderated the relationship between cognitive flexibility and advice network centrality.

The negative coefficient of the interaction term indicated that the relationship between cognitive flexibility and advice network centrality became more negative as self-monitoring increased. However, it is not easy to determine the size and precise nature of this effect by examining the coefficients alone. This prompted the authors to plot the effect using a simple slope test to calculate predicted values of advice network centrality under different conditions (a common method to use values that are one SD above and below the mean to show high and low values of the cognitive flexibility and high and low values of self-monitoring) and predicted relationship between the cognitive flexibility and advice network centrality at different levels of self-monitoring (Dawson, 2014).

**FIGURE 1:**
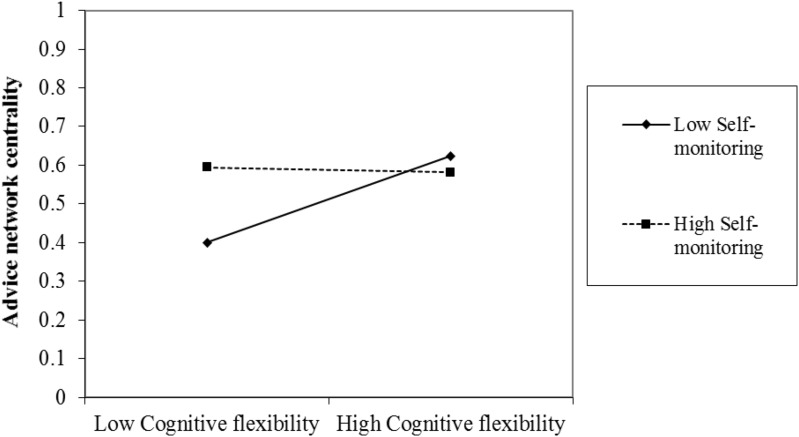
The moderating effect of self-monitoring on the relationship between cognitive flexibility and advice network centrality.

The simple slope test result showed that cognitive flexibility had a significant positive effect on advice network centrality when self-monitoring was low (β = 0.117, *t* = 2.976, and *p* < 0.01), whereas cognitive flexibility had a non-significant positive effect on advice network centrality when self-monitoring was high (β = -0.007, *t* = -0.592, and *p* = 0.56). **Figure 1** illustrates the interaction effect of self-monitoring and cognitive flexibility on advice network centrality: when an individual’s cognitive flexibility moved from low to high, the low self-monitor’s advice network centrality increased sharply and significantly; however, the high self-monitor’s advice network centrality did not increase. This result suggested that the positive effect of cognitive flexibility on the advice network centrality only exists for low self-monitors, but not for high self-monitors.

## Discussion

This study aimed to explore the individual differences in gaining central positions in workplace advice network. The results demonstrated that the more cognitively flexible a person was, the more central his/her position in the advice network. Furthermore, self-monitoring mitigated this positive effect of cognitive flexibility on occupying advice network centrality. Specifically, cognitive flexibility was positively associated with advice network centrality only for low self-monitoring individuals. It implies that cognitively flexible individuals who monitor less of their public self-image in interpersonal relationships achieve higher level of network centrality. In contrast, cognitively flexible individuals who monitor more of their public self-image in interpersonal relationships possess have no impact on occupying advice network centrality. These findings advanced the understanding of how workplace social structure can be shaped by personality traits and how individuals get the central position in advice networks.

### Theoretical Implications

This study has three theoretical implications. First, this study contributed to the literature of individual differences in obtaining a central position in an advice network by exploring a cognitive factor. Previous research finds that individual differences in demographic characteristics, personality, and helping behaviors could predict advice network centrality (Klein et al., 2004; Balkundi et al., 2011; Erdogan et al., 2015). This study demonstrated that cognitive flexibility is another predictor that could explain why some individuals are sought-after advice sources. Moreover, this study also discovered self-monitoring as a cue for advice seekers to evaluate the accessibility of the value of a potential adviser’s cognitive ability. That is, unlike low self-monitoring individuals, high self-monitoring individuals might be unable to obtain their network status advantage despite of their high cognitive ability. A possible explanation for this effect is that low self-monitors tend to exhibit consistent behaviors which in turn make others perceived themselves as not only capable but also accessible and reliable.

Second, this study contributed to cognitive flexibility literature by examining a novel consequential variable – an individual’s position in workplace advice network. Cognitively flexible individuals exhibit higher level of creativity (Chen et al., 2014) and higher resilience to stress or negative events (Genet and Siemer, 2011). This study extended the downstream consequences of cognitive flexibility from behavioral variables to a structural property in social network, by showing that cognitive flexibility can help people obtain higher status in their advice network.

Third, this study also contributed to literature related to self-monitoring. Consistent with previous study that uncover the negative influence of self-monitoring (Bhardwaj et al., 2015), our paper revealed that self-monitoring impaired the positive influence of cognitive flexibility on advice network centrality. These outcomes also support previous research that demonstrates a moderating role of self-monitoring (Barrick et al., 2005).

### Practical Implications

Practical implications can be drawn from these findings for employees and leaders. The research identifies people’s tendency to seek advice from individuals who can yield more benefits (being high cognitive flexible) and incur less costs (being low self-monitoring) in social interaction. Hence, employees who lack formal power in the workplace could gain informal power by exhibiting their cognitive flexibility during interpersonal interactions. Moreover, for leaders who want to promote advice-seeking behaviors among team members, the implication is that the advice source role does not favor people with low cognitive flexibility and high self-monitoring.

### Limitations and Future Research

Despite the efforts made in this research, there are limitations that could be improved in future research. First, the two time-points survey design of this study was correlational. Future research could consider using an experimental method to manipulate cognitive flexibility. Second, this study focuses on the effect of cognitive flexibility on advice network centrality in the context of salesmen. Future research could extend the findings to more general settings that feature a high frequency of social interactions, such as sports team. Third, since it takes time to evaluate the value of advice seeking action, future research could explore whether the advice seeker’s future time orientation may play a moderating role. With regards to the effect of cognitive flexibility on advice network centrality only holds for low self-monitors in this study, further research could investigate the mechanism of individuals’ behavior consistency and reliability.

## Author Contributions

SH and XZ designed the study and wrote the manuscript. JZ collected and analyzed the data. ZZ revised the manuscript. All authors contributed to the interpretation of the results and final version of the paper.

## Conflict of Interest Statement

The authors declare that the research was conducted in the absence of any commercial or financial relationships that could be construed as a potential conflict of interest.
